# *In vitro* activity of cefepime/zidebactam against sulbactam/durlobactam-susceptible and -resistant *Acinetobacter baumannii* clinical isolates

**DOI:** 10.1093/jac/dkaf467

**Published:** 2026-01-19

**Authors:** Carlo Tascini, Gabriele Bianco, Robert A Bonomo, Paolo Gaibani

**Affiliations:** Department of Medicine, Università degli Studi di Udine, Udine, Italy; Infectious Diseases Unit, Azienda Sanitaria Universitaria Friuli Centrale, Udine, Italy; Department of Experimental Medicine, University of Salento, Lecce, Italy; Microbiology and Virology Unit, Vito Fazzi Hospital, Lecce, Italy; Department of Population and Quantitative Health Sciences, School of Medicine, Case Western Reserve University, Cleveland, USA; Department of Diagnostic and Public Health, Microbiology Section, Verona University, Verona, Italy; Microbiology and Virology Unit, Azienda Ospedaliera Universitaria Integrata Di Verona, Verona, Italy

## Abstract

**Objectives:**

To evaluate the *in vitro* activity of cefepime in association with a β-lactamase inhibitor (enmetazobactam) or β-lactam enhancer (BLE), zidebactam, against carbapenem-resistant *Acinetobacter baumannii* (CRAB) strains susceptible or resistant to sulbactam/durlobactam.

**Material and methods:**

Twenty-one CRAB clinical isolates were characterized by WGS and AST to cefepime/enmetazobactam, cefepime/zidebactam and comparators was determined.

**Results:**

Resistome analysis revealed that all CRAB carried *bla*_OXA-23_ carbapenemase genes, while *bla*_ADC-25_ and *bla*_OXA-66_ β-lactamase genes were observed exclusively in sulbactam/durlobactam-resistant strains. Analysis of penicillin-binding protein genes demonstrated the presence of specific mutations within PBP3 (N392T) previously associated to the resistance to sulbactam/durlobactam. Phenotypic analysis revealed that cefepime/enmetazobactam did not exert antibacterial activity against CRAB, while cefepime/zidebactam displayed potent bactericidal activity against both sulbactam/durlobactam-susceptible and -resistant strains.

**Conclusions:**

These results demonstrate that cefepime/zidebactam exhibited potent *in vitro* antibacterial activity against CRAB producing OXA carbapenemase and support its clinical use against both sulbactam/durlobactam-susceptible or -resistant isolates.

## Introduction

*Acinetobacter baumannii* is an opportunistic Gram-negative pathogen and a leading cause of hospital-acquired infections, particularly in critically ill and immunocompromised patients. Historically considered ‘low virulence’, resistant strains are now associated with mortality rates up to 70%.^1,2^ The bacterium rapidly acquires antimicrobial resistance via mobile genetic elements, upregulation of efflux pumps, β-lactamases, aminoglycoside-modifying enzymes and target gene mutations.^3^ The global emergence of MDR, XDR and PDR strains represents a major clinical challenge.^3,4^

Carbapenem-resistant *A. baumannii* (CRAB) is frequently cross-resistant to last-line agents such as colistin and tigecycline, and has been classified by the WHO as an urgent public health threat, highlighting the need for novel therapeutic options.^5^ Sulbactam/durlobactam is a recently approved combination for CRAB infections. Sulbactam, a β-lactamase inhibitor with intrinsic antibacterial activity, binds penicillin-binding proteins, whereas durlobactam, a novel diazabicyclooctane (DBO) β-lactamase inhibitor similar to avibactam, protects sulbactam from degradation by serine β-lactamases, particularly OXA-type enzymes.^6,7^ This combination demonstrated clinical efficacy in HABP and VABP in the ATTACK trial and is currently recommended by the Infectious Diseases Society of America Guidance as a preferred option for CRAB infections, often in combination with a carbapenem.^7,8^ Resistance has been reported via PBP mutations and metallo-β-lactamase expression.^9^

Among recently developed agents are two cefepime-based β-lactam/β-lactamase inhibitor combinations. Cefepime, a fourth-generation cephalosporin, is bactericidal through PBP inhibition and resistant to most AmpC β-lactamases but hydrolysed by ESBLs. Cefepime/enmetazobactam combines cefepime with enmetazobactam, a β-lactamase inhibitor similar to tazobactam that protects cefepime from ESBL-mediated degradation.^10,11^

Cefepime/zidebactam is another novel combination, where zidebactam binds PBP2 and cefepime targets PBP3, producing rapid bactericidal activity, even at sub-MIC levels, independently of β-lactamase expression.^12^ Zidebactam exerts its activity by dual mechanism of action by enhancing the activity of the β-lactams partner.^12^ In detail, zidebactam inhibits class A, C and some D B-lactamase, and directly binds to Penicillin-Binding Protein 2 (PBP2) in Gram-negative bacteria, thus enhancing the killing of *Enterobacterales* and *Pseudomonas aeruginosa* and producing KPC or MBL when combined with cefepime.^13^

However, a significant discrepancy in susceptibility was observed in carbapenem-resistant and/or carbapenemase-producing *A. baumannii*, with rates of 95.7% using the provisional PK/PD breakpoint (≤64 mg/L) versus 24.9% with the CLSI cefepime breakpoint (≤8 mg/L).^12^

The aim of the present study is to evaluate the *in vitro* activity of cefepime/enmetazobactam and cefepime/zidebactam against CRAB isolates characterized by WGS, including strains susceptible and resistant to sulbactam/durlobactam.

## Material and methods

### Bacteria characterization

The CRAB isolates were selected from two Italian strain collections from university hospitals in Northern Italy and consisted of *Acinetobacter baumannii* bloodstream isolates from hospitalized patients. The selection comprised all consecutive blood culture isolates collected in 2022–2023 that were resistant to sulbactam/durlobactam. In addition, seven consecutive isolates susceptible to sulbactam/durlobactam were included. Duplicate CRAB isolates collected from the same patient were excluded. Antimicrobial susceptibility testing was performed using Vitek2 system (Biomerieux, France) and results were confirmed by SensititreTM Plate EUMDRXXF (Thermofisher, USA). MICs for cefiderocol were assayed by microdilution in iron depleted-Mueller–Hinton broth with the ComASP cefiderocol test (Liofilchem, Italy), while MICs for sulbactam/durlobactam, cefepime/enmetazobactam and cefepime/zidebactam were evaluated by MIC TestStrip (Liofilchem, Roseto degli Abruzzi, Italy). All MIC values were determined in triplicate. *Escherichia coli* ATCC 25922 and *Pseudomonas aeruginosa* ATCC 27853 were used as quality control strains to validate antimicrobial susceptibility testing. Results were interpreted following EUCAST and CLSI clinical breakpoints (for cefepime/zidebactam, the provisional PK/PD susceptibility breakpoint was >64 mg/L). Comparison between MICs of different antibiotics was performed using Student’s *t*-test analysis implemented in GraphPad Prism v.10.1.11 (San Diego, CA, USA).

### Genome-sequencing analysis

Genomic DNA were extracted from purified bacterial cultures of *A. baumannii* using the DNeasy Blood&Tissue Kit (Qiagen, Switzerland) and cleaned up with AMPure XP magnetic beads (Beckman Coulter). Bacterial genomes were sequenced as previously described.^14^ Briefly, libraries were prepared with DNA Prep Library Preparation Kit (Illumina, USA) and sequenced by the Illumina MiSeq platform (Illumina, USA) using the MiSeq Reagent Kit v.3 with 2 × 300 paired-end reads. The quality of the reads was evaluated using FastQC v.12.1 software (hps://www.bioinformatics.babraham.ac.uk/projects/fastqc/, accessed on 1 January 2023) and assembly was performed using SPAdes v.3.10. Antimicrobial resistance determinants and MLST analysis were evaluated using an online platform (available at https://www.genomicepidemiology.org/). The phylogenetic trees based on core genomes were generated as previously described.^14^ Analysis of PBPs were performed by aligning gene sequences against reference genome of ATCC17978 *A. baumannii* strain using ClustalW.^14^ SNP detection was manually curated using Unipro UGENE v.49.1.

## Results

Genomic characteristics of the CRAB clinical isolates included in the study are shown in the Table 1. Analysis of antimicrobial determinants related to β-lactams-resistance showed that all isolates also carried *bla*_OXA-23_, while 61.9% (13/21), 47.6% (10/21) and 14.2% (3/21) carried *bla*_ADC-25_, *bla*_OXA-66_ and *bla*_NDM-1_ β-lactamase genes, respectively. These genetic antimicrobial determinants were observed only in the sulbactam/durlobactam-resistant strains. Notably, 75% (6/8) of the sulbactam/durlobactam-susceptible CRAB carried *bla*_TEM-1_.

**Table 1.** Genotypical characteristics of carbapenem-resistant *Acinetobacter baumannii* (CRAb) clinical strains included in this study

Strain	Β-lactamases	Other resistance determinants	PBP mutations				
			PBP1a	PBP1b	PBP2	PBP3	PBP5
CRAb16	*bla*_OXA-69_, *bla*_ADC-25_, *bla*_OXA-23_ *bla*_NDM-1_	*aph(6)-Id, aph(3*″*)-Ib, aph(3*′*)-Ia, sul2*	T38A, A244T, T776A	N513H	P665A	L480I, T511S	WT
CRAB45	*bla*_OXA-66_, *bla*_ADC-25_, *bla*_OXA-23_	*aph(6)-Id, aph(3*″*)-Ib, sul2, tet(B)*	WT	P112S	WT	A515V	N329S
CRAB55	*bla*_OXA-66_, *bla*_ADC-25_, *bla*_OXA-23_	*aph(6)-Id, aph(3*″*)-Ib, sul2, tet(B)*	WT	P112S, G137R	WT	N392T	N329S
CRAB59	*bla*_OXA-66_, *bla*_ADC-25_, *bla*_OXA-23_	*aph(6)-Id, aph(3*″*)-Ib, sul2, tet(B)*	WT	P112S, G137R	WT	N392T	N329S
CRAB66	*bla*_OXA-66_, *bla*_ADC-25_, *bla*_OXA-23_	*aph(6)-Id, aph(3*″*)-Ib, sul2, tet(B)*	WT	P112S, G137R	WT	N392T	N329S
CRAB68	*bla*_OXA-66_, *bla*_ADC-25_, *bla*_OXA-23_	*aph(6)-Id, aph(3*″*)-Ib, sul2, tet(B)*	WT	P112S, G137R	WT	N392T	N329S
CRAB75	*bla*_OXA-66_, *bla*_ADC-25_, *bla*_OXA-23_	*aph(6)-Id, aph(3*″*)-Ib, sul2, sul1, tet(B)*	WT	P112S, G137R	WT	N392T	N329S
CRAB19	*bla*_OXA-69_, *bla*_ADC-25_, *bla*_OXA-23_ *bla*_NDM-1_	*aph(6)-Id, aph(3*″*)-Ia, armA, sul1, tet(B)*	T38A, A244T, T776A	N513H	P665A	L480I, T511S	WT
CRAB28	*bla*_OXA-69_, *bla*_ADC-25_, *bla*_OXA-23_ *bla*_NDM-1_	*ant(2*″*)-Ia, armA, aac(3*′*)-Ia, aph(3*′*)-Ia, tet(B)*	T38A, A244T, T776A	N513H	P665A	L480I, T511S	WT
CRAB48	*bla*_OXA-66_, *bla*_ADC-25_, *bla*_OXA-23_	*aph(6)-Id, aph(3*″*)-Ib, armA, sul2, tet(B)*	A184V	P112S, G137R	WT	N392T	N329S
CRAB35	*bla*_OXA-66_, *bla*_ADC-25_, *bla*_OXA-23_	*aph(6)-Id, aph(3*″*)-Ib, sul2, tet(B)*	WT	P112S, G137R	WT	N392T	N329S
CRAB93	*bla*_OXA-66_, *bla*_ADC-25_, *bla*_OXA-23_	*aph(6)-Id, aph(3*″*)-Ib, sul2, tet(B)*	WT	P112S	WT	N392T	N329S
CRAB96	*bla*_OXA-66_, *bla*_ADC-25_, *bla*_OXA-23_	*aph(6)-Id, aph(3*″*)-Ib, armA, sul2, tet(B)*	WT	P112S	WT	N392T	N329S
CRAB10_BO	*bla_TEM1D_*, *bla*_OXA-23_	*armA, aph(3*″*)-Ib, aph(3*″*)-Ia*	WT	P112S	WT	A515V	N329S
CRAB11_BO	*bla*_OXA-23_	*armA, aph(3*″*)-Ib, tet(B), sul2*	WT	P112S	WT	A515V	N329S
CRAB12_BO	*bla_TEM1D_*, *bla*_OXA-23_	*armA, aph(3*″*)-Ib, aph(3*″*)-Ia, tet(B), sul2*	WT	P112S	WT	A515V	N329S
CRAB24_BO	*bla_TEM1D_*, *bla*_OXA-23_	*armA, aph(3*″*)-Ib, aph(3*″*)-Ia, tet(B),*	WT	P112S	WT	A515V	N329S
CRAB29_BO	*bla*_OXA-23_	*armA, aph(3*″*)-Ib, tet(B), sul2*	WT	P112S	WT	A515V	N329S
CRAB30_BO	*bla_TEM1D_*, *bla*_OXA-23_	*armA, aph(3*″*)-Ib, aph(3*″*)-Ia, tet(B)*	WT	P112S	WT	A515V	N329S
CRAB34_BO	*bla_TEM1D_*, *bla*_OXA-23_	*armA, aph(3*″*)-Ib, aph(3*″*)-Ia, tet(B)*	WT	P112S	WT	A515V	N329S
CRAB38_BO	*bla_TEM1D_*, *bla*_OXA-23_	*armA, aph(3*″*)-Ib, aph(3*″*)-Ia, tet(B)*	WT	P112S	WT	A515V	N329S

WT, wild type.

Analysis of genes related to resistance to other antimicrobial class showed that 90.5% (19/21) carried *aph(3*″*)-Ib*, 71.4% (15/21) carried *sul1/2* or tetB and 57.1% (12/21) carried *aph(6)-Id*. Analysis of PBPs sequences revealed all strains harboured a similar allelic profile (i.e. wild type, PBP1a; P112S, PBP1b; wild type, PBP2; N392S, PBP5) excluding NDM-producing CRAB isolates. At the same time, specific mutations were observed within *PBP3* gene between sulbactam/durlobactam-resistant (90%, N392T) and -susceptible (100%, A515V) strains. In addition, exclusive mutations within PBP genes were observed among NDM-producing CRAB [i.e. *PBP1a* (T38A, A244T and T766A); *PBP1b* (N513H); *PBP2* (P665A); *PBP3* (L480I, T511S)].

To evaluate the genomic relatedness of CRAB strains included in the study, phylogenetic analysis based on core-genome SNPs was performed. The derived phylogenetic tree showed that the isolates included in the study were related to other CRAB strains isolated in Italy, while strains carrying *bla*_NDM-1_ segregated separately to other Italian strains (Figure S1, available as Supplementary data at *JAC* Online in the Supplementary material).

Phenotypic characteristics of the CRAB clinical isolates included in the study are shown in the Table S1 (in the Supplementary Material). Phenotypic characteristics of the CRAB clinical isolates included in the study are shown in Table S1 (in the Supplementary Material). Phenotypic results showed that all CRAB strains included in this study exhibited high MIC values for meropenem (median >16 mg/L, IQR 16 mg/L), cefepime (median >64 mg/L, IQR 64 mg/L) and cefepime/enmetazobactam (median >64 mg/L), whereas they showed low MICs for cefiderocol (median 0.5 mg/L, IQR 0.125–1 mg/L). At the same time, the median MIC for sulbactam/durlobactam against susceptible strains was 2 mg/L (IQR 2 mg/L), while it was 64 mg/L (IQR 64 mg/L) against resistant CRAB strains.

**Figure 1.**
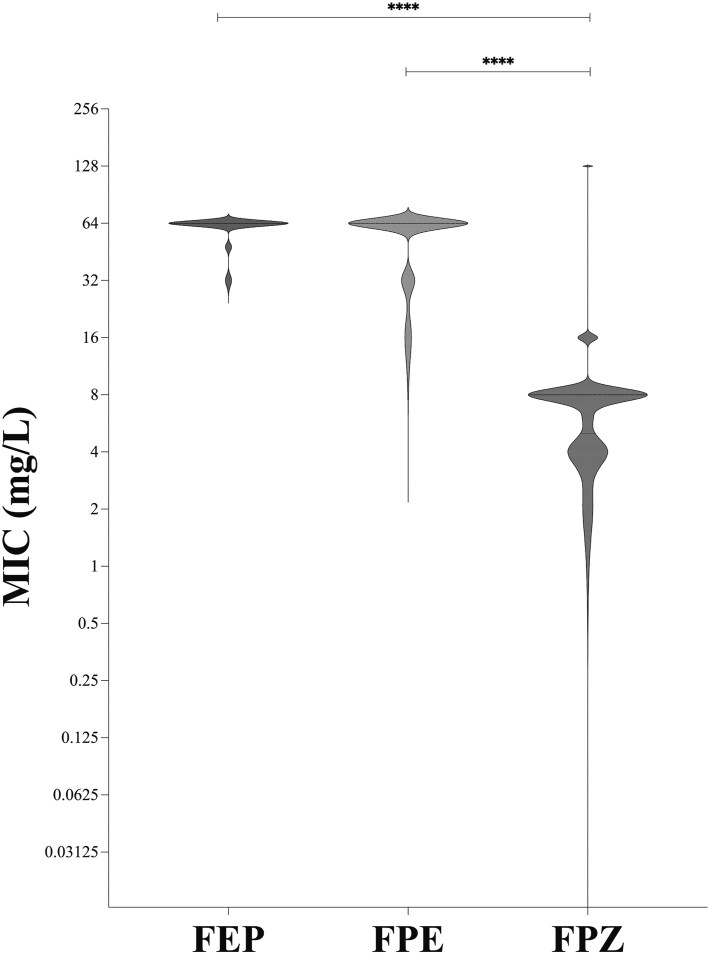
MIC of cefepime, cefepime/enmetazobactam and cefepime/zidebactam against CRAB clinical strains included in this study.

Of note, our results showed that cefepime/zidebactam demonstrated potent antibacterial activity against all CRAB strains included in the study (Figure 1) by showing a significant reduction in MICs (*P* < 0.0001) in comparison to cefepime and cefepime/enmetazobactam. In addition, our results showed that cefepime/zidebactam exerted higher antibacterial activity against sulbactam/durlobactam-susceptible strains (median 8 IQR 6.5–8; *P* < 0.0001) than sulbactam/durlobactam-resistant strains (median 8 IQR 4–8; *P* < 0.01) (Figure S2 in the Supplementary material).

## Discussion

Here we showed that cefepime/zidebactam displayed a high antibacterial effect against CRAB strains including sulbacatam/durlobactam-susceptible and -resistant clinical strains. Our results are partially in agreement with data presented in the literature showing a broad range of MICs of cefepime/zidebactam against *A. baumannii* clinical strains.^15–18^ In particular, our results reveal that the MIC_90_ for cefepime/zidebactam was 8 mg/L against CRAB strains included in this study, and similar results were observed by Mushtaq and co-workers against acquired OXA carbapenemases strains and Sader *et al*. against imipenem-susceptible strains *A. baumannii* clinical isolates.^15,16^ Of note, we observed that statistical differences were not observed between sulbactam/durlobactam-susceptible and -resistant strains. These findings are in accordance with genotypic results by showing the absence of mutations within the main target of zidebactam (i.e. PBP2) among CRAB strains included in the study, thus resulting in a similar antibacterial activity against these two groups independent of the sulbactam/durlobactam resistance. We also hypothesized that the differences in cefepime/zidebactam activity against sulbactam/durlobactam-susceptible and -resistant strains is probably caused by the different β-lactamase gene contents (i.e. *bla*_ADC-25_, *bla*_OXA-66_ β-lactamase genes present in sulbactam/durlobactam-resistant strains) in association with specific mutations within the PBP-1 and PBP-3, which are targets of cefepime.^17^ A recent review highlighted resistance mechanisms to cefepime/zidebactam in Gram-negative bacteria, suggesting that resistance may involve multiple mutations in PBPs, as well as alterations in efflux systems.^12^ In this context, the combination of efflux pump alterations, β-lactamase expression and PBP mutations could explain the high MIC observed in the CRAB48 isolate.

In addition, we observed that enmetazobactam did not increased the activity of cefepime against CRAB strains independent of the reduced susceptibility to sulbactam/durlobactam. These results are in agreement with previous studies showing the absence of inhibitory activity of cefepime/enmetazobactam against *A. baumannii* independent of antimicrobial resistance genes carried by isolates.^18,19^

Here we provided the *in vitro* evidence of the antibacterial activity of cefepime/zidebactam against sulbactam/durlobactam-susceptible and -resistant CRAB strains. In this context, a recent study showed that human-simulated exposure of cefepime-zidebactam determined a significant bactericidal effect against carbapenem-resistant *A. baumannii* expressing OXA carbapenemases in the murine thigh infection model, thus suggesting *in vivo* efficacy of this combination.^20^

In conclusion, our findings suggest that cefepime/zidebactam may have potential activity against infections caused by carbapenem-resistant *A. baumannii*, including both sulbactam/durlobactam-susceptible and -resistant strains, and could represent a potential candidate for the treatment of CRAB infections. However, this study has some limitations. The generalizability of our findings is limited by the relatively small number of bacterial strains included and the fact that isolates were collected from only two Italian centres. Larger multicentre studies, ideally including global strain collections, are needed to confirm and extend these findings and to better define the clinical impact of cefepime/zidebactam for the treatment of infections caused by sulbactam/durlobactam-resistant CRAB.

## Supplementary Material

dkaf467_Supplementary_Data

## Data Availability

Data supporting this study will be available upon reasonable request.

## Supplementary data

Figures S1 and S2 and Table S1 are available as Supplementary data at *JAC* Online.
