# Supplementary material for: In vitro activity of cefepime/zidebactam against sulbactam/durlobactam-susceptible and -resistant Acinetobacter baumannii clinical isolates
Source: J Antimicrob Chemother. 2026 Jan 19;81(2):dkaf467. doi: 10.1093/jac/dkaf467 (PMC12813283; doi:10.1093/jac/dkaf467)
Supplement: dkaf467_Supplementary_Data [file dkaf467_supplementary_data.docx]

**Table S1.** MICs of Cefepime (FEP), Cefepime/enmetazobactam (FPE), Cefepime/Zidebactam (FDZ), Sulbactam/Durlobactam (SUL/DUR), Cefiderocol (CFD) and Meropenem (MRP) against Carbapenem-Resistant *Acinetobacter baumannii* (CRAB) clinical strains included in this study.

|  | **MIC**  **(mg/L)** | | | | | |
| --- | --- | --- | --- | --- | --- | --- |
|  | **FEP** | **FPE** | **FPZ** | **SUL/DUR** | **FDC** | **MRP** |
| CRAB16 | >64 | >64 | 8 | >64 | 8 | >16 |
| CRAB45 | >64 | >64 | 4 | >64 | 1 | >16 |
| CRAB55 | >64 | >64 | 8 | >64 | 1 | >16 |
| CRAB59 | >64 | 32 | 8 | >64 | 0.5 | >16 |
| CRAB66 | 64 | 64 | 8 | >64 | 1 | >16 |
| CRAB68 | 48 | 16 | 16 | >64 | 0.5 | >16 |
| CRAB75 | 32 | 32 | 8 | >64 | 0.25 | >16 |
| CRAB19 | >64 | >64 | 4 | >64 | 4 | >16 |
| CRAB28 | >64 | >64 | 2 | >64 | 4 | >16 |
| CRAB48 | >64 | >64 | 128 | >64 | 1 | >16 |
| CRAB35 | >64 | >64 | 8 | >64 | 8 | >16 |
| CRAB93 | >64 | >64 | 8 | >64 | 1 | >16 |
| CRAB96 | >64 | >64 | 4 | >64 | 0.25 | >16 |
| CRAB10_BO | >64 | >64 | 8 | 2 | 0.125 | >16 |
| CRAB11_BO | >64 | >64 | 6 | 2 | 0.125 | >16 |
| CRAB12_BO | >64 | >64 | 4 | 2 | 0.125 | >16 |
| CRAB24_BO | >64 | >64 | 8 | 2 | 0.25 | >16 |
| CRAB29_BO | >64 | >64 | 8 | 1 | 0.125 | >16 |
| CRAB30_BO | >64 | >64 | 8 | 2 | 0.125 | >16 |
| CRAB34_BO | >64 | >64 | 8 | 2 | 0.064 | >16 |
| CRAB38_BO | >64 | >64 | 16 | 4 | 0.25 | >16 |

**Figure S1.** Phylogenetic tree based on core-genome SNPs among Carbapenem-Resistant *Acinetobacter baumannii* (CRAB) clinical strains included in this study





**Figure S2.** MIC of cefepime, cefepime/enmetazobactam and cefepime/zidebactam against sulbactam/durlobactam -susceptible (panel A) and -resistant (panel B) Carbapenem-Resistant *Acinetobacter baumannii* clinical strains included in this study.
